# Reliable diagnosis of nigrostriatal degeneration by dopamine transporter SPECT despite drug interaction with venlafaxine or bupropion

**DOI:** 10.1007/s00259-024-06989-z

**Published:** 2024-11-30

**Authors:** Ivayla Apostolova, Sabine Hellwig, Amir Karimzadeh, Susanne Klutmann, Philipp T. Meyer, Ralph Buchert

**Affiliations:** 1 Department of Nuclear Medicine, University Medical Center Hamburg-Eppendorf, Martinistr. 52, 20246 Hamburg, Germany; 2 Department of Psychiatry and Psychotherapy, Medical Center - University of Freiburg, Freiburg, Germany; 3 Department of Nuclear Medicine, Medical Center - University of Freiburg, Freiburg, Germany

**Keywords:** Dopamine transporter, [^123^I]FP-CIT, Drug interaction, Venlafaxine, Bupropion

## Abstract

**Purpose:**

This study examined the impact of venlafaxine and bupropion on the detection of nigrostriatal degeneration by dopamine transporter (DAT)-SPECT.

**Methods:**

43 patients (70.7 ± 8.6y, 44% female) with [^123^I]FP-CIT-SPECT under venlafaxine (*n* = 26, 37.5-225 mg/d), bupropion (*n* = 16, 150 or 300 mg/d) or both (*n* = 1) were included retrospectively. The striatal specific [^123^I]FP-CIT binding ratio (SBR), its left–right asymmetry and the putamen-to-caudate ratio were transformed to z-scores and submitted to a cluster analysis for data-driven categorization.

**Results:**

Two clusters were identified. The first cluster (37% cases) showed a Parkinson’s disease (PD)-like pattern: median striatal SBR/asymmetry/putamen-to-caudate z-score -4.5/4.9/-3.8. The second cluster (63%) showed symmetric reduction with normal intra-striatal gradient: median striatal SBR/asymmetry/putamen-to-caudate z-score -2.7/0.4/0.2. Patients with follow-up clinical reference diagnoses of neurodegenerative (*n* = 8) and non-neurodegenerative (*n* = 16) parkinsonism fell exclusively into the former or the latter cluster, respectively (*p* < 0.001).

**Conclusion:**

Venlafaxine and bupropion cause uniform reduction of the striatal [^123^I]FP-CIT SBR that can be distinguished from PD-like reductions.

**Supplementary Information:**

The online version contains supplementary material available at 10.1007/s00259-024-06989-z.

## Introduction

Depression is a major comorbidity in Parkinson’s disease [1]. Thus, a relevant proportion of patients referred to dopamine transporter (DAT)-SPECT with N-ω-fluoropropyl-2β-carbomethoxy-3β-(4-I-123-iodophenyl)nortropane ([^123^I]FP-CIT) receives antidepressants including the norepinephrine-dopamine reuptake inhibitor (NDRI) bupropion and venlafaxine, usually considered a serotonin-norepinephrine reuptake inhibitor (SNRI) [2].

There is a relative lack of studies on the impact of the latter antidepressants on [^123^I]FP-CIT-SPECT [3–7]. As a consequence, current practice guidelines are somewhat inconsistent regarding the necessity of their withdrawal before [^123^I]FP-CIT-SPECT. The joint EANM/SNMMI guideline recommends withdrawal of bupropion but not venlafaxine prior to [^123^I]FP-CIT-SPECT [8], whereas the German guideline recommends to discontinue both, venlafaxine and bupropion [9]. These recommendations and their discrepancy are of high clinical relevance, as even short-term withdrawal of antidepressants can be critical in severely depressed patients. Particularly, withdrawal of venlafaxine may cause several somatic as well as severe (neuro)psychiatric symptoms [10].

This retrospective study examined the impact of venlafaxine and bupropion on the utility of [^123^I]FP-CIT-SPECT to detect nigrostriatal degeneration.

## Materials and methods

### Patients

The databases of the University Medical Centers of Freiburg and Hamburg were searched for clinical [^123^I]FP-CIT-SPECT performed on persistent venlafaxine or bupropion treatment. Patients who had withdrawn the drug in preparation of [^123^I]FP-CIT-SPECT or had skipped the single dose on the morning of the SPECT examination were excluded. There were no further eligibility criteria. This resulted in the inclusion of 43 patients (70.7 ± 8.6y, 44% female): 26 under venlafaxine (37.5–225 mg/d), 16 under bupropion (150 or 300 mg/d), one patient under both (112.5/150 mg/d venlafaxine/bupropion).

A reference diagnosis based on clinical follow-up and/or FDG- and/or tau- and/or amyloid-PET and/or repeated [^123^I]FP-CIT-SPECT after drug withdrawal was available in 24 patients: neurodegenerative/non-neurodegenerative parkinsonism (with/without nigrostriatal degeneration) in 8/16 (33/67%) patients.

Demographical and clinical data are given in Table 1.

**Table 1:** Demographical and clinical data. Age and drug dose are given as mean±standard deviation (range)

	All	Venlafaxine	Bupropion	Both
Number of patients	43	26	16	1
Age	70.7 ± 8.6 (52–89)	72.7 ± 8.3 (52–89)	68.3 ± 8.1 (55–83)	57.8
Sex (% female)	44	54	25	
Collimator (% LEHR)	51	27	87	100
Venlafaxine dose (mg/d)	115 ± 66 (37.5–225) n = 27	115 ± 67 (37.5–225)		112.5
Bupropion dose (mg/d)	259 ± 70 (150 or 300) n = 11		270 ± 63 (150 or 300) n = 10	150
Reference diagnosis (non-neurodegenerative/neurodegenerative/ not available)	16/8/19	4/5/17	11/3/2	1/0/0

### SPECT imaging

DAT-SPECT was performed after intravenous injection of about 180 MBq [^123^I]FP-CIT on a triple-head camera equipped with multiple-pinhole collimators [11] (*n* = 21) or on a double- or triple-head camera equipped with parallel-hole low-energy-high-resolution (LEHR) collimators (*n* = 22).

The multiple-pinhole projection data were reconstructed with the Monte Carlo photon simulation engine and iterative one-step-late maximum-a-posteriori expectation–maximization implemented in the camera software (24 iterations, 2 subsets) [11]. Chang’s method was used for attenuation correction.

The LEHR projection data were reconstructed using iterative ordered-subsets-expectation–maximization with attenuation and simulation-based scatter correction as well as collimator-detector response modelling implemented in the Hybrid Recon-Neurology tool of the Hermes SMART workstation v1.6 (5 iterations, 15/16 subsets for 120/128 views, 7 mm gaussian postfiltering).

### Semi-quantitative analyses

Semi-quantitative analyses were performed as described previously [11]. In brief, individual [^123^I]FP-CIT-SPECT images were spatially normalized to the Montreal Neurological Institute space using the Normalize tool of the Statistical Parametric Mapping software package (SPM12) and a set of custom target [^123^I]FP-CIT-SPECT images (representative of normal striatal signal and different levels of PD-like reductions). Semi-quantitative distribution volume ratio (DVR) images were obtained by voxelwise scaling to the individual 75th percentile of the voxel intensity in a reference region comprising the whole brain without striata, thalamus, medial temporal lobe, brainstem, cerebellum, and ventricles.

The unilateral [^123^I]FP-CIT specific binding ratio (SBR) of left and right caudate, putamen and whole striatum was obtained by hottest voxels analysis of the spatially normalized DVR image using large unilateral masks. The masks were much bigger than the corresponding anatomical structures in order to guarantee that the entire structure was completely included into the mask in each individual subject, independent of some residual anatomical between-subjects variability after spatial normalization. The number of hottest voxels used for the DVR estimation was defined by the typical volume of the respective anatomical structure in healthy subjects [12]: 10 ml for the unilateral putamen (1250 cubic voxels of 2 mm edge length), 5 ml for the unilateral caudate (625 voxels) and 15 ml for the unilateral striatum (1875 voxels). The SBR of left and right caudate, putamen and striatum were obtained as SBR = DVR–1. Mean striatum SBR and putamen-to-caudate ratio were obtained by averaging the respective measures of both hemispheres. The asymmetry of the striatum SBR was computed as asymmetry[%] = 200*abs(left–right)/(left + right).

The SBR in the serotonin transporter (SERT)-rich midbrain was estimated from the same DVR images by averaging the voxel intensities across the midbrain mask provided by the TD Lobes atlas implemented in the WFU-PickAtlas.

In order to eliminate systematic differences between multiple-pinhole and LEHR data, each of the aforementioned semi-quantitative measures was transformed to z-scores relative to the corresponding mean and standard deviation in a normative reference database. This was performed separately for multiple-pinhole and LEHR data. Both normative databases were built from consecutive clinical [^123^I]FP-CIT-SPECT without any eligibility criteria except that the images had been interpreted as normal. Image reconstruction, preprocessing and semi-quantitative analyses in the normative databases were performed exactly as in the patients under venlafaxine and/or bupropion. The MPH normative database comprised 39 DAT-SPECT (mean patient age 68.0 ± 15.0y, 51% female), the LEHR normative database comprised 49 DAT-SPECT (68.3 ± 10.9y, 53% female). Neither normative database differed from the corresponding patients under venlafaxine and/or bupropion regarding age (*p* ≥ 0.249) or sex (*p* ≥ 0.412).

### Statistical analysis

For visual assessment of the [^123^I]FP-CIT-SPECT, DVR images were presented in randomized order independently to two experienced readers (≥ 12 years, ≥ 3,000 cases). A fixed upper threshold on the color table was used [13]. The readers were asked to categorize the images into one of three categories: “normal” versus “PD-like reduction” versus “uniform reduction”.

Fully data-driven categorization of the 43 [^123^I]FP-CIT-SPECT regarding their striatal signal was performed using the two-step cluster analysis implemented in IBM SPSS (version 29) with default parameter settings (distance measure: log-likelihood probability distribution, clustering criterion: Schwarz’s Bayesian information criterion, determination of the number of clusters: automatic). The three striatal z-scores (striatal specific binding ratio, its left–right asymmetry, putamen-to-caudate ratio) were used as independent (continuous) predictor variables. The [^123^I]FP-CIT-SPECT were ordered randomly prior to the cluster analysis in order to avoid order effects.

A 2 × 2 contingency table and the chi-squared test were used to test the clustering for an association with the reference diagnosis.

The mean striatum SBR z-score was tested for correlation with the drug dose using the non-parametric Spearman rank-order test. This was done separately for venlafaxine and bupropion and separately in each cluster from the cluster analysis.

All *p*-values are given two-sided. Statistical significance was assumed if *p* < 0.05.

## Results

**Fig. 1:**
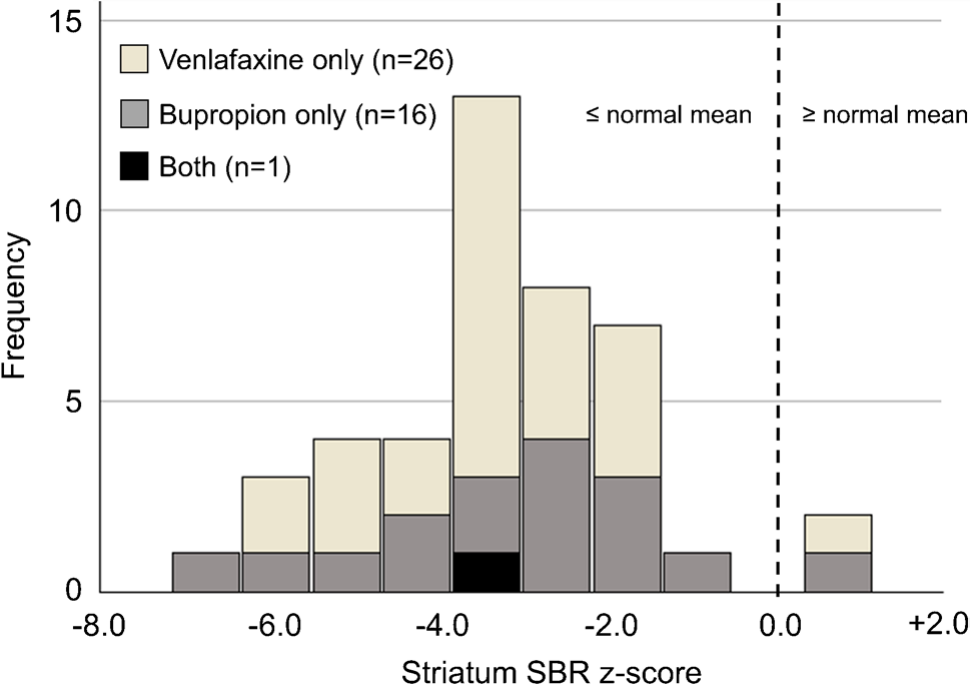
Histogram of the z-score of the specific binding ratio (SBR) of [^123^I]FP-CIT in the bilateral striatum

The distribution of the striatal SBR among the 43 patients with [^123^I]FP-CIT-SPECT under venlafaxine, bupropion or both is shown in Fig. 1. Thirty-five (81%) patients showed moderate or more severe reduction of the striatal signal (striatum SBR z-score < −2), 5 (12%) patients showed mild reduction (−1 > z-score ≥ −2).

**Fig. 2:**
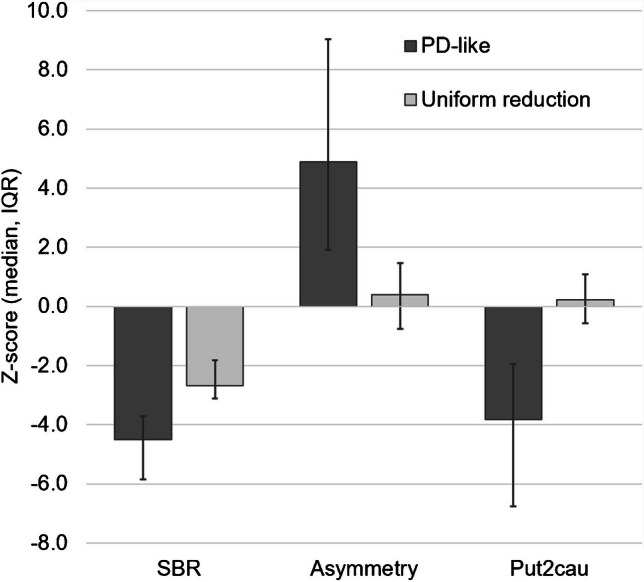
Z-score of the mean striatum specific binding ratio (SBR), its left–right asymmetry and the mean putamen-to-caudate ratio according to cluster membership. The bars indicate median values, the error bars interquartile ranges (IQR)

**Fig. 3:**
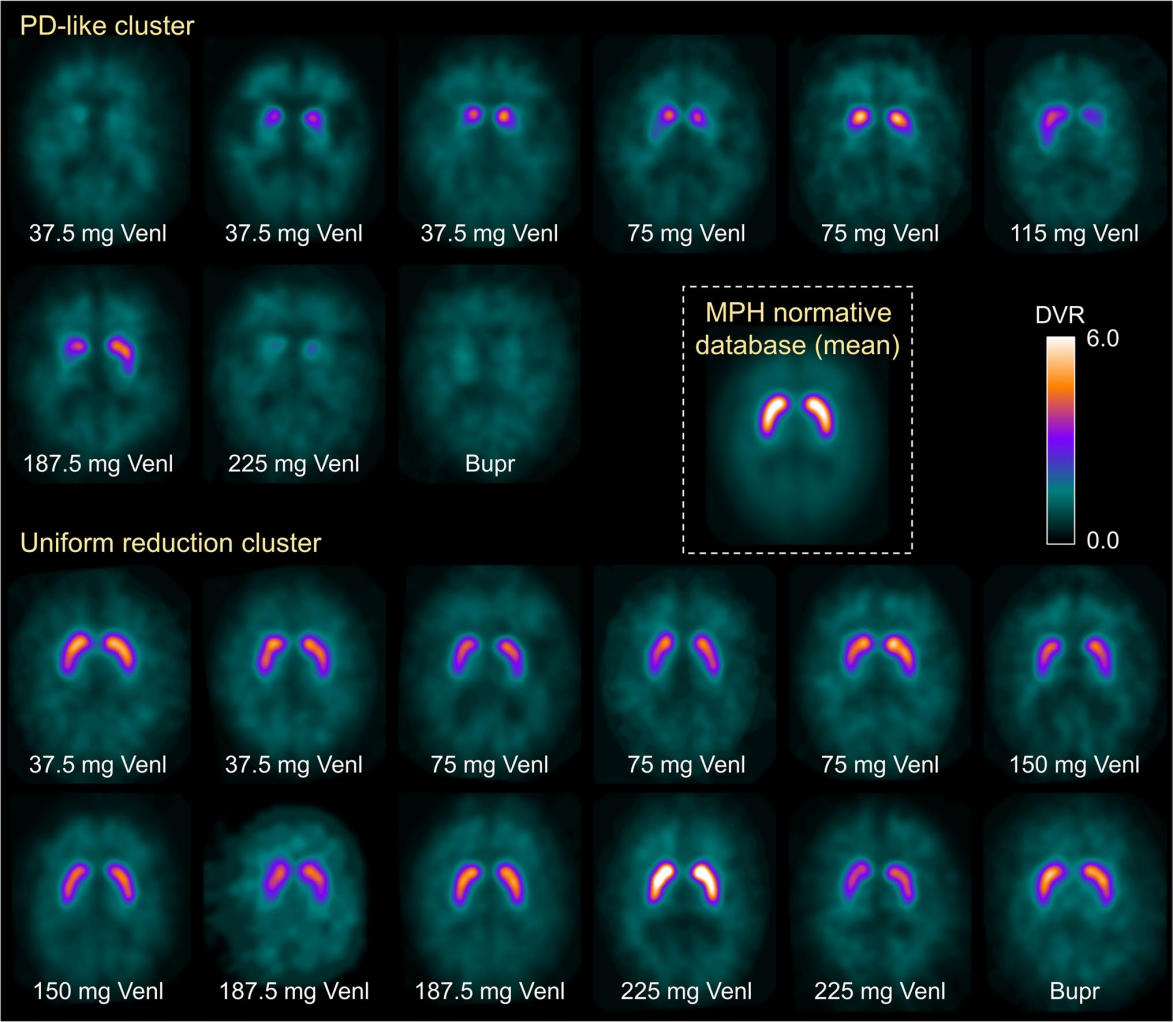
[^123^I]FP-CIT distribution volume ratio (DVR) images acquired under venlafaxine (Venl) or bupropion (Bupr) with multiple-pinhole collimators. The DVR images are separated according to cluster membership. The insert shows the voxel-wise mean of the 39 [^123^I]FP-CIT -SPECT in the multiple-pinhole normal database. All images are shown with the same thresholds on the color table

The data-driven cluster analysis identified two clusters of good quality (Silhouette measure > 0.5) and without outliers. The first cluster comprised 16 cases (37%) and was characterized by a PD-like pattern: striatal binding was markedly reduced (median striatal SBR z-score: −4.5, [interquartile range: −5.8,−3.7]), showed high hemispheric asymmetry (median asymmetry z-score: 4.9 [1.9,9.0]) and a steep rostro-caudal gradient (median putamen-to-caudate z-score: −3.8 [−6.8,−2.0]). This cluster is denoted as “PD-like” cluster. The remaining 27 cases (63%) were included in the second cluster characterized by less pronounced, though still notable reduction of striatal binding (median SBR z-score: −2.7 [−3.1,−1.8]). Striatal binding was symmetric (median asymmetry z-score: 0.4 [−0.8,1.5]) and showed a normal rostro-caudal gradient (median putamen-to-caudate z-score: 0.2 [−0.6,1.1]) (Fig. 2). This cluster is denoted as “uniform reduction” cluster. The two clusters did neither differ in age (Mann–Whitney U test *p* = 0.209) nor in sex (Pearson chi-square test *p* = 0.965). The automatic categorization of the multiple-pinhole images is summarized in Fig. 3.

Discrepancy in the visual categorization between the two readers was observed in 2 (5%) of the 43 cases (discrepancy regarding the discrimination between “normal” and “uniform reduction” in both cases). The readers were asked to reach a consensus in these cases. The contingency table of the consensus visual read versus the automatic categorization by the cluster analysis is shown in Table 2. A relevant discrepancy between the visual read and the cluster analysis was observed in 3 (7%) of the 43 cases (Supplementary Fig. 1).

**Table 2:** Contingency table of the consensus visual read versus the automatic categorization by the cluster analysis

		Visual consensus read		
		Normal	Uniform reduction	PD-like reduction
Cluster analysis	Uniform reduction	7	18	2
	PD-like reduction	0	1	15

All patients with a reference diagnosis of neurodegenerative parkinsonism (*n* = 8) were automatically categorized into the PD-like cluster, all patients with follow-up diagnosis of non-neurodegenerative parkinsonism (*n* = 16) were automatically categorized into the uniform reduction cluster (Pearson chi-square test *p* < 0.001).

**Fig. 4:**
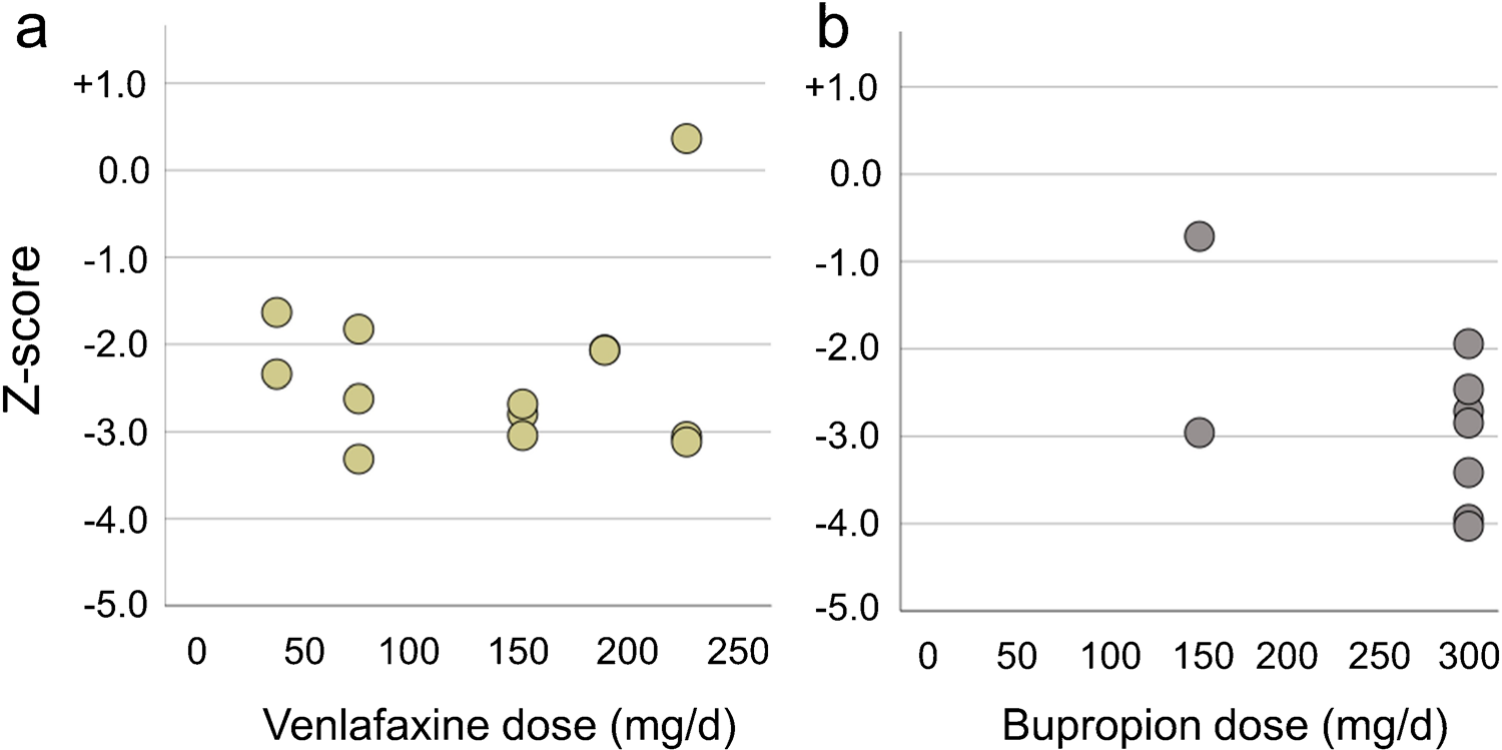
Striatum specific binding ratio (SBR) z-score versus venlafaxine (**a**) or bupropion (**b**) dose in the uniform reduction cluster

The test for correlation between the mean striatum SBR and the drug dose in the uniform reduction cluster did not reveal a significant effect, neither in the patients under venlafaxine (*n* = 13, rho = −0.165, *p* = 0.589, Fig. 4a) nor in the patients under bupropion (*n* = 9, rho = –0.311, *p* = 0.416, Fig. 4b).

Repeat [^123^I]FP-CIT-SPECT after withdrawal of bupropion or venlafaxine are shown in Supplementary Fig. 2.

The SBR in the SERT-rich midbrain was systematically reduced in the venlafaxine group (independent of the cluster membership), supporting that [^123^I]FP-CIT SPECT was actually performed under the SNRI venlafaxine (*n* = 26, median z-score −2.8, interquartile range [−3.3, −2.3]). In the bupropion group, the midbrain SBR was close to normal (*n* = 16, median z-score –0.4 [−1.8, + 0.2]), as expected for the NDRI bupropion.

## Discussion

The findings of this study are as follows. First, persistent treatment with typical doses of venlafaxine or bupropion is associated with a reduction of the striatal [^123^I]FP-CIT SBR that can be similar in magnitude to the reduction caused by nigrostriatal degeneration.

Second, venlafaxine-/bupropion-induced reduction of the [^123^I]FP-CIT SBR is rather uniform throughout both striata, that is, left–right symmetric and without PD-like rostro-caudal gradient. As a consequence, discrimination between venlafaxine-/bupropion-induced reduction and nigrostriatal degeneration is feasible, either by using cluster analysis (more or less independent of the method used for the SBR analysis, Supplementary Fig. 3) or by visual inspection. The observed discrepancy between the visual read and the cluster analysis in 7% of the cases is in line with the rate of 5–10% “inconclusive” cases in clinical [^123^I]FP-CIT SPECT that cannot be classified as either “normal” or “reduced” with acceptable certainty even by expert readers [14, 15]. Critical consideration is required in patients suspected to be at an early stage of dementia with Lewy bodies or an atypical neurodegenerative parkinsonian syndrome, since these might be associated with more uniform nigrostriatal degeneration. Furthermore, old age might also contribute to uniform reduction of the striatal signal in [^123^I]FP-CIT SPECT, as the age-related decline of DAT availability is very similar in caudate and putamen, both in women and men [16]. In all these cases, drug withdrawal (if clinically justifiable) or study repetition after withdrawal (in case of questionable studies without withdrawal) is advisable. Uniform reduction of the striatal signal has also been observed by Hsiao and co-workers performing DAT-SPECT with ^99m^Tc-TRODAT-1 before and after 8 weeks of bupropion treatment in 23 patients with major depression (without parkinsonism) [6].

Third, the uniform reduction of the striatal [^123^I]FP-CIT SBR caused by typical doses is similar for venlafaxine and bupropion. A recent systematic review of the potential effects of medications and drugs of abuse on [^123^I]FP-CIT-SPECT concluded that bupropion treatment causes a 14–25% decrease of striatal [^123^I]FP-CIT binding and recommended discontinuing bupropion for 5 days [3]. The same review did not find sufficient evidence to recommend withdrawal of venlafaxine [3], mainly based on a prospective study in 8 healthy controls showing 10.7 ± 3.0% increase of the striatal [^123^I]β-CIT SBR after 4 days with 75 mg followed by 5 days with 150 mg venlafaxine [4]. However, there is a report of a false positive DAT-SPECT under 225 mg venlafaxine, which was resolved by a repeat scan after venlafaxine withdrawal [5]. The current study clearly supports this case report and suggests that venlafaxine treatment causes a reduction of the striatal [^123^I]FP-CIT SBR similar to bupropion. This finding is surprising given that the affinity for the DAT is at least one order of magnitude higher for bupropion compared with venlafaxine (K_D_ = 520 versus 9300 nM [17]). Thus, non-specific or serotonin transporter-mediated effects of venlafaxine on [^123^I]FP-CIT uptake appear more likely than direct DAT blocking. Finally, we consider it highly unlikely that the consistent venlafaxine-/bupropion-associated uniform reduction observed in non-PD patients is an effect of depression, since depression-related DAT changes are at most very small [18].

Fourth, no significant relationship between the dose and the striatal SBR was observed, neither for venlafaxine nor for bupropion. This might be explained by the rather small sample sizes in the uniform reduction cluster (n = 13 and n = 9 for venlafaxine and bupropion, respectively). Furthermore, there might be some selection bias: assuming that the antidepressant dose was systematically increased in each subject until a therapy effect occurred, between-subjects variability of receptor occupancy might have been small in the included patients despite considerable between-subjects variability of the dose.

The current study included data acquired with LEHR or multiple-pinhole collimators. We expect that the findings apply to all settings that fulfill the joint EANM/SNMMI recommendation that “given the small structures in the basal ganglia, reconstructed SPECT spatial resolution should be ≤ 10 mm full-width-at-half-maximum” [8].

Limitations of the current study include the rather small sample size and its retrospective nature. The latter explains that a reference diagnosis was available in only half of the included patients. Most of the remaining patients had been referred to [^123^I]FP-CIT SPECT in our departments by external neurology practices so that we did not have access to these patients’ files to check their clinical follow-up. The lack of a clinical reference diagnosis in these patients most likely does not present a relevant selection bias for this study. The available clinical reference diagnoses were taken from the patients’ files and, therefore, might be biased by the [^123^I]FP-CIT SPECT findings. However, the clinical reference diagnosis was supported by repeat [^123^I]FP-CIT SPECT (in *n* = 5 patients, Supplementary Fig. 2) and/or [^18^F]FDG-PET (*n* = 15) and/or tau-PET with [^18^F]PM-PBB3 (n = 2) and/or amyloid-PET with [^11^C]PIB (*n* = 1) in 16 (67%) of the 24 patients. The median duration of the clinical follow-up in the remaining 8 patients (without support by additional nuclear medicine imaging procedures) was 8 months (interquartile range 6-15 months).

In conclusion, venlafaxine and bupropion cause uniform reduction of the striatal [^123^I]FP-CIT signal that differs from PD-like patterns. Nevertheless, withdrawal before [^123^I]FP-CIT-SPECT should be preferred.

## Supplementary Information

Below is the link to the electronic supplementary material.

Supplementary file1 (DOCX 1.72 MB)

## Data Availability

Clinical data (age, sex, venlafaxine and bupropion dose) and z-scores derived from the [^123^I]FP-CIT-SPECT images are available from the corresponding author on reasonable request.
