# Supplementary material for: Reliable diagnosis of nigrostriatal degeneration by dopamine transporter SPECT despite drug interaction with venlafaxine or bupropion
Source: Eur J Nucl Med Mol Imaging. 2024 Nov 30;52(4):1549–55. doi: 10.1007/s00259-024-06989-z (PMC11839892; doi:10.1007/s00259-024-06989-z)
Supplement: Supplementary file 1 — Supplementary file1 (DOCX 1.72 MB) [file 259_2024_6989_MOESM1_ESM.docx]

**Supplementary Information**

**Reliable diagnosis of nigrostriatal degeneration by dopamine transporter SPECT despite drug interaction with venlafaxine or bupropion**

Ivayla Apostolova^1,*^, Sabine Hellwig^2,*^, Amir Karimzadeh^1^, Susanne Klutmann^1^, Philipp T. Meyer^3,**^, Ralph Buchert^1,**^

^1^Department of Nuclear Medicine, University Medical Center Hamburg-Eppendorf, Hamburg, Germany

^2^Department of Psychiatry and Psychotherapy and ^3^Department of Nuclear Medicine, Medical Center - University of Freiburg, Freiburg, Germany

^*^These authors contributed equally as first authors

^**^These authors contributed equally as last authors

**Corresponding author** (not in training): Ralph Buchert, Department of Nuclear Medicine, University Medical Center Hamburg-Eppendorf, Martinistr. 52, 20246 Hamburg, Germany, Email: r.buchert@uke.de, Phone: +49-(0)40-7410-54347, Fax: +49-(0)40-7410-40265, ORCID-ID 0000-0002-0945-0724

**Supplementary figures**

**
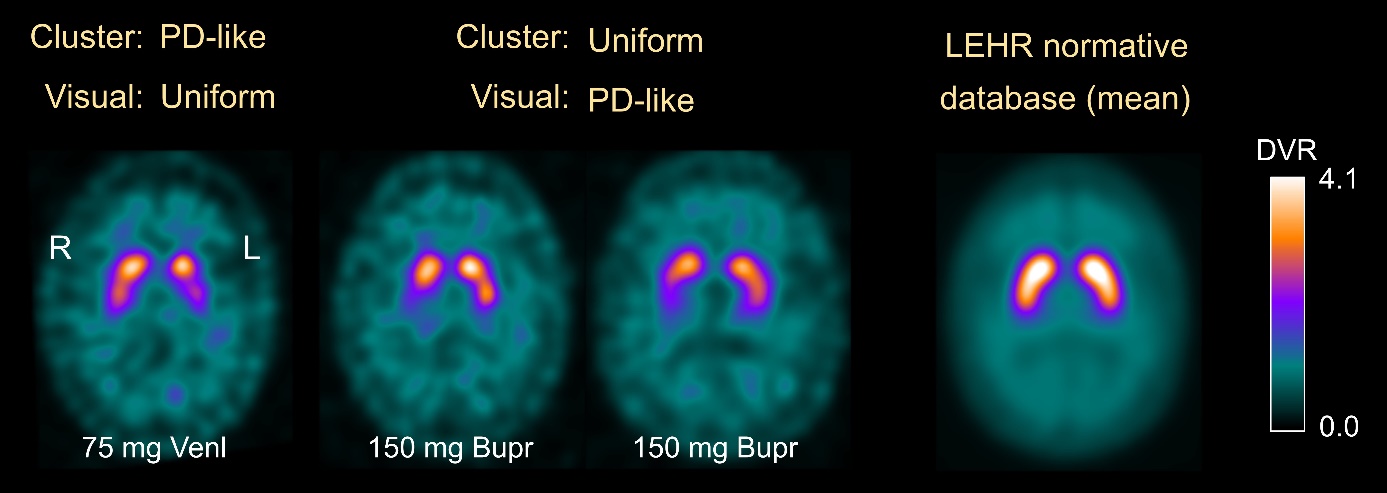
**

**Supplementary Fig. 1** [^123^I]FP-CIT distribution volume ratio (DVR) images acquired under venlafaxine (Venl) or bupropion (Bupr) with discrepancy between the automatic cluster analysis and visual interpretation regarding their categorization (PD-like reduction versus uniform reduction). The [^123^I]FP-CIT SPECT were acquired with LEHR collimator. The image on the right shows the voxel-wise mean of the 49 [^123^I]FP-CIT -SPECT in the LEHR normal database for comparison. All images are shown with the same thresholds on the color table.

**
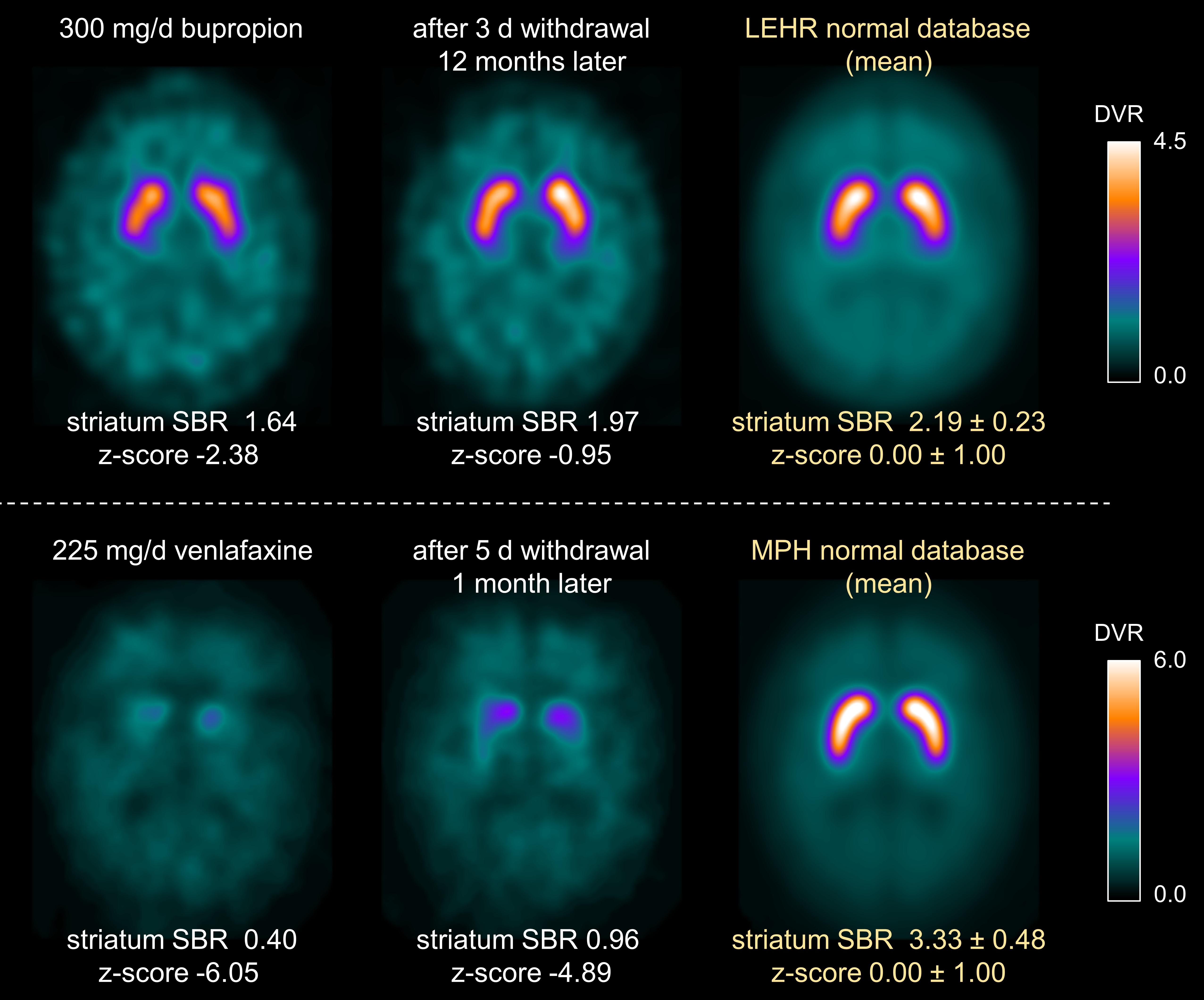
**

**Supplementary Fig. 2** Repeat [^123^I]FP-CIT-SPECT after 3 days withdrawal of 300 mg/d bupropion (top) and after 5 days withdrawal of 225 mg/d venlafaxine (bottom). The [^123^I]FP-CIT-SPECT under bupropion was automatically categorized into the uniform reduction cluster, the [^123^I]FP-CIT-SPECT under venlafaxine was automatically categorized into the PD-like cluster. Repeat [^123^I]FP-CIT-SPECT were acquired with the same camera used at baseline (top: double-head camera with LEHR collimators, bottom: triple-head camera with multiple-pinhole collimators). The voxel-wise mean image in the corresponding normative reference database is shown for comparison.


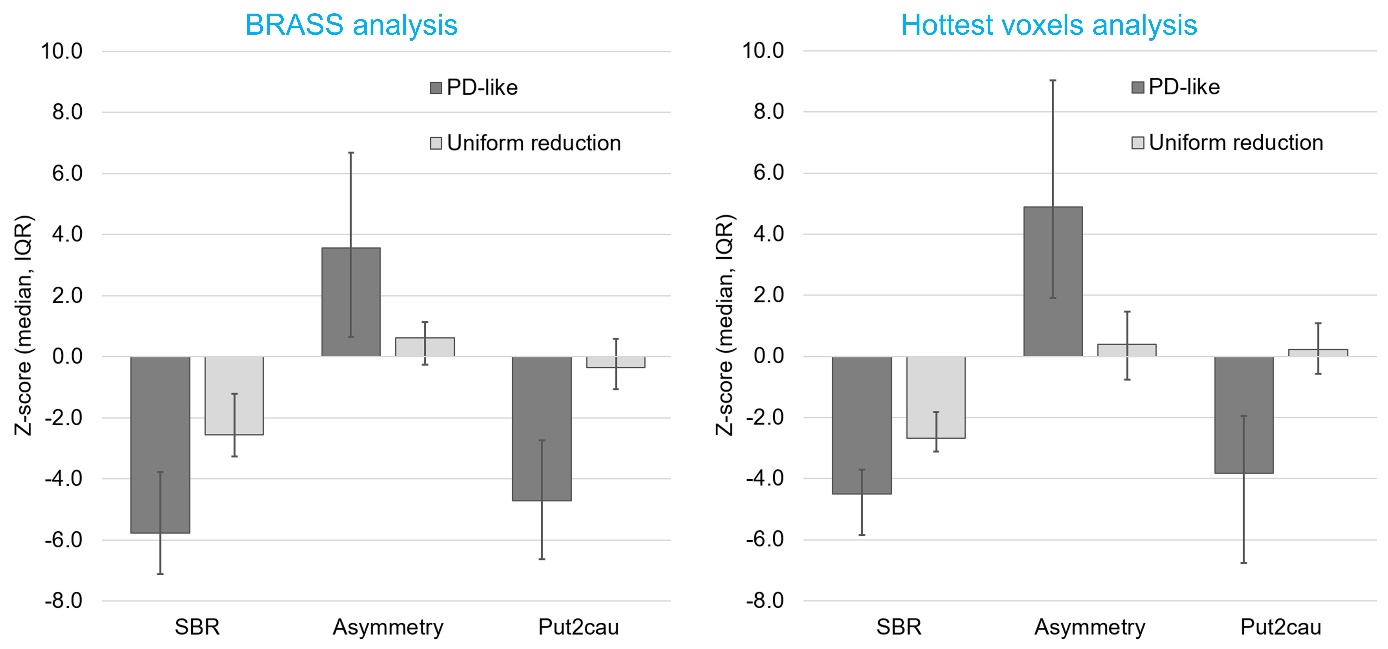


**Supplementary Fig. 3** Z-score of the mean striatum specific binding ratio (SBR), its left-right asymmetry and the mean putamen-to-caudate ratio according to cluster membership. The bars indicate median values, the error bars interquartile ranges (IQR). For the semi-quantitiative “BRASS analysis” (left), the unilateral [^123^I]FP-CIT SBR of left and right caudate, putamen and whole striatum was obtained with the BRASS tool for [^123^I]FP-CIT SPECT implemented in the Hermes SMART workstation (version 1.6) with default parameter settings (reference region: occipital cortex). The BRASS analysis was performed for all scans under venlafaxine and/or bupropion and for all scans in both normative databases. Then, each of the three semi-quantitative measures was transformed to z-scores relative to the corresponding mean and standard deviation of the BRASS estimates in the corresponding normative reference database. For comparison, the z-scores from the hottest voxels analysis described in the manuscript are shown on the right (same as Figure 2 in the manuscript). The clusters (PD-like, uniform reduction) were identified by the two-step cluster analysis of the z-scores from the hottest voxels analysis.
